# Recombination, decreased host specificity and increased mobility may have driven the emergence of maize streak virus as an agricultural pathogen

**DOI:** 10.1099/vir.0.2008/003590-0

**Published:** 2008-09

**Authors:** Arvind Varsani, Dionne N. Shepherd, Adérito L. Monjane, Betty E. Owor, Julia B. Erdmann, Edward P. Rybicki, Michel Peterschmitt, Rob W. Briddon, Peter G. Markham, Sunday Oluwafemi, Oliver P. Windram, Pierre Lefeuvre, Jean-Michel Lett, Darren P. Martin

**Affiliations:** 1 Electron Microscope Unit, University of Cape Town, Rondebosch, Cape Town 7701, South Africa; 2 Department of Molecular and Cell Biology, University of Cape Town, Rondebosch, Cape Town 7701, South Africa; 3 Institute of Biology, Department of Molecular Biology and Plant Virology, University of Stuttgart, Pfaffenwaldring 57, D-70550 Stuttgart, Germany; 4 Institute of Infectious Disease and Molecular Medicine, University of Cape Town, Anzio Road, Observatory, Cape Town 7925, South Africa; 5 CIRAD, UMR BGPI, TA A54/K, Campus International de Baillarguet, 34398 Montpellier Cedex 5, France; 6 National Institute for Biotechnology and Genetic Engineering, Jhang Road, PO Box 577, Faisalabad, Pakistan; 7 Department of Disease and Stress Biology, John Innes Centre, Norwich NR4 7UH, UK; 8 Department of Crop, Soil and Environmental Management, Bowen University, PMB 284, Iwo, Osun State, Nigeria; 9 Warwick HRI Biology Centre, University of Warwick, Wellesbourne CV35 9EF, UK; 10 CIRAD, UMR 53 PVBMT CIRAD-Université de la Réunion, Pôle de Protection des Plantes, Ligne Paradis, 97410 Saint Pierre, La Réunion, France

## Abstract

Maize streak virus (MSV; family *Geminiviridae*, genus *Mastrevirus*), the causal agent of maize streak disease, ranks amongst the most serious biological threats to food security in subSaharan Africa. Although five distinct MSV strains have been currently described, only one of these – MSV-A – causes severe disease in maize. Due primarily to their not being an obvious threat to agriculture, very little is known about the ‘grass-adapted’ MSV strains, MSV-B, -C, -D and -E. Since comparing the genetic diversities, geographical distributions and natural host ranges of MSV-A with the other MSV strains could provide valuable information on the epidemiology, evolution and emergence of MSV-A, we carried out a phylogeographical analysis of MSVs found in uncultivated indigenous African grasses. Amongst the 83 new MSV genomes presented here, we report the discovery of six new MSV strains (MSV-F to -K). The non-random recombination breakpoint distributions detectable with these and other available mastrevirus sequences partially mirror those seen in begomoviruses, implying that the forces shaping these breakpoint patterns have been largely conserved since the earliest geminivirus ancestors. We present evidence that the ancestor of all MSV-A variants was the recombinant progeny of ancestral MSV-B and MSV-G/-F variants. While it remains unknown whether recombination influenced the emergence of MSV-A in maize, our discovery that MSV-A variants may both move between and become established in different regions of Africa with greater ease, and infect more grass species than other MSV strains, goes some way towards explaining why MSV-A is such a successful maize pathogen.

## INTRODUCTION

Maize streak virus (MSV; family *Geminiviridae*, genus *Mastrevirus*) is best known as the causal agent of maize streak disease. Although the geographical range of MSV is largely restricted to subSaharan Africa, its serious impact on food security in the world's poorest countries ranks it amongst the most important agricultural pathogens globally (Bosque-Perez, 2000).

Although a significant degree of MSV diversity has been documented (Dekker *et al.* 1988; Clarke *et al.*, 1989; Pinner & Markham, 1990; Peterschmitt *et al.*, 1991; Martin *et al.*, 2001; Willment *et al.*, 2001), only one low diversity strain, called MSV-A (McClean, 1947; Storey & McClean, 1930), is responsible for maize streak disease (Pinner *et al.*, 1988; Briddon *et al.*, 1994). MSV-A variants are generally the only viruses sampled from field-collected maize plants presenting with severe streak disease, and the only variants known to cause the disease under laboratory conditions (Martin *et al.*, 2001).

Other strains of MSV – called MSV-B to -E, but often collectively referred to as ‘grass-infecting’ or non-maize-adapted MSVs – have only very rarely been isolated from maize plants, and generally only produce very mild symptoms in even the most MSV-sensitive maize genotypes (Pinner *et al.*, 1988; Martin *et al.*, 1999, 2001; Schnippenkoetter *et al.*, 2001; Willment *et al.* 2002). While this might suggest that these other MSV strains are largely irrelevant to African agriculture, they have been found infecting other cultivated crops such as wheat, rye, barley and oats (Willment *et al.*, 2001). Under laboratory testing conditions, MSV-B and -C are significantly more virulent than MSV-A isolates in wheat and barley (Schnippenkoetter *et al.*, 2001; Willment *et al.*, 2002), suggesting that these viruses may have a substantial, albeit an as yet undetermined, impact on African agriculture.

Besides their potential agricultural relevance, the non-maize-adapted MSV strains may hold important clues to the past and ongoing evolution and epidemiology of the maize-adapted MSV-A strain. Given that the most prevalent MSV-A variant in southern Africa – MSV-A_4_ – is actually a MSV-A/B recombinant (Martin *et al.*, 2001), there is a clear precedent for the non-maize-adapted MSV strains directly contributing via recombination to ongoing MSV-A evolution. More thorough analyses of MSV recombination involving a greater diversity of MSV full-length genomes could, as has been done with other geminiviruses (Lefeuvre *et al.*, 2007b; Prasanna & Rai, 2007), provide a more detailed picture of sequence exchange patterns most commonly associated with evolutionary advancement of MSVs. Also, from both an ecological and evolutionary perspective, comparative analyses of geographical and host range distributions of different MSV strains could help identify unique aspects of MSV-A epidemiology that have facilitated its emergence as an important agricultural pathogen.

We therefore undertook a survey of MSV diversity in indigenous uncultivated African grass species. Amongst 83 MSV isolates sampled in West Africa, East Africa, southern Africa and La Réunion, we identified six new MSV strains. We present evidence of extensive inter-strain MSV recombination and identify recombination breakpoint hot- and cold-spots that are partially conserved amongst all geminiviruses. Our analysis reveals significant differences in the natural host and geographical ranges of various MSV strains that may have a bearing on the emergence of MSV-A as a maize pathogen.

## METHODS

### Virus sampling.

Grasses displaying symptoms characteristic of MSV infection were sampled between 2005 and 2007 from South Africa (*n*=39), Zimbabwe (*n*=1), Mozambique (*n*=4), Nigeria (*n*=9), La Réunion (*n*=8) and Uganda (*n*=7). We also analysed archived samples collected in previous studies between 1986 and 2000 from Nigeria (*n*=3), Burundi (*n*=1) and Rwanda (*n*=1; Pinner *et al.*, 1988; Pinner & Markham, 1990), Mali (*n*=1), Zimbabwe (*n*=2; Peterschmitt *et al.*, 1991) and South Africa (*n*=7; Willment *et al.*, 2001). Only one sample was taken per host species in any given sampling location and different sampling locations were separated by two or more kilometres (see Supplementary Table S1 available in JGV Online for sampling coordinates, dates and host species).

### Cloning and sequencing of full genomes.

Viral genomes were isolated from plant material as described previously (Owor *et al.*, 2007a; Shepherd *et al.*, 2008a). Amplified concatemers were digested with either *Bam*HI, *Kpn*I or *Sal*I to yield ∼2.7 kb linearized viral genomes that were ligated into pGEMZf+ (Promega Biotech). Both strands of cloned genomes were commercially sequenced (Macrogen) using the primer set described by Owor *et al.* (2007a).

### Host species identification.

Host species were identified by chloroplast *ndhF* gene sequencing. C-terminal encoding portions (∼ 1.1 kb) of the *ndhF* genes were amplified from grass genomic DNA using the PCR primers: 972-F (5′-GTCTCAATTGGGTTATATGATG-3′) and 2110-R (5′-CCCCCTAYATATTTGATACCTTCTCC-3′) using Kapa *Taq* (Kapa Biosystems) described by Olmstead & Sweere (1994) and Giussani *et al.* (2001). The *ndhF* amplicons were ligated into pGEM-T Easy (Promega Biotech) and completely sequenced by Macrogen using M13 standard sequencing primers.

### Sequence analyses.

All available African streak virus genome sequences, including those of *Urochloa* streak virus (Oluwafemi *et al.*, 2008), Eragrostis streak virus (Shepherd *et al.*, 2008b), *Panicum* streak virus (Briddon *et al.*, 1992; Varsani *et al.*, 2008), Sugar cane streak virus (Hughes *et al.*, 1993; Shepherd *et al.*, 2008b), Sugar cane streak Egypt virus (Bigarré *et al.*, 1999) and Sugar cane streak Reunion virus (Bigarré *et al.*, 1999; Shepherd *et al.*, 2008b), were obtained from public sequence databases. Sequence alignments were constructed using the poa program (Grasso & Lee, 2004) and edited both by eye and using the clustal w-based (Thompson *et al.*, 1994) sequence alignment tool implemented in mega (version 4; Tamura *et al.*, 2007). mega was also used to calculate the pairwise sequence identities shared by aligned genomes using pairwise deletion of gaps.

Maximum-likelihood phylogenetic trees were constructed using the phyml program (Guindon & Gascuel, 2003). The F81+G_4_ nucleotide substitution model was selected as being the most appropriate for the analysis of MSV evolution using the modeltest web server (Posada, 2006)

Recombination was analysed using the rdp (Martin & Rybicki, 2000), geneconv (Padidam *et al.*, 1999), bootscan (Martin *et al.*, 2005a), maxchi (Smith, 1992), chimaera (Posada & Crandall, 2001), siscan (Gibbs *et al.*, 2000) and 3seq (Boni *et al.*, 2007) methods implemented in the rdp3 program (Martin *et al.*, 2005b). Default settings were used throughout and only potential recombination events detected by two or more of the above methods, coupled with phylogenetic evidence of recombination, were considered significant. The severity of Bonferroni correction was minimized by only searching for recombination signals in a single sequence within groups of sequences sharing >99.3 % sequence identity. Using the approach outlined in the rdp3 program manual (http://darwin.uvigo.es/rdp/rdp.html), the approximate breakpoint positions and recombinant sequence(s) inferred for every potential recombination event were manually checked and adjusted where necessary using the phylogenetic and recombination signal analysis features available in rdp3.

The distribution of unambiguously detected breakpoint positions of all unique recombination events was analysed for evidence of recombination hot- and cold-spots with rdp3 as described by Heath *et al.* (2006). Published rdp3 project files describing breakpoint distributions detectable in bipartite and monopartite begomoviruses (Lefeuvre *et al.*, 2007b) were merged in rdp3 and used to produce a composite plot of begomovirus recombination breakpoint distributions.

## RESULTS AND DISCUSSION

### Discovery of new MSV strains

We cloned and fully sequenced 83 individual MSV genomes sampled primarily from indigenous African grasses presenting with streak symptoms. For preliminary objective classification of these sequences we aligned them with a selection of MSV (*n*=88) and non-MSV (*n*=24) African streak virus genomes and determined pair-wise percentage sequence identities shared between them. All of the new sequences shared greater than 79.1 % identity with previously described MSV isolates (Supplementary Table S2 available in JGV Online) and, based on the current ICTV species demarcation guidelines for the mastreviruses (Stanley *et al.*, 2005), they are all MSV strain isolates.

Consistent with previous analyses of African streak virus diversity (Martin *et al.*, 2001; Willment *et al.*, 2002; Shepherd *et al.*, 2008b; Varsani *et al.*, 2008), we further subdivided the sequences into strain groupings. As is clearly indicated by a deep trough between 92 and 94 % identity in a plot of pair-wise MSV sequence identities (Supplementary Fig. S1 available in JGV Online) and, in accordance with Martin *et al.* (2001), we identified 93 % identity as a ‘natural’ MSV strain demarcation threshold. Using this criterion we classified the MSV isolates into 11 strains (named MSV-A to -K), only five of which (MSV-A to -E) have been described previously (Martin *et al.*, 2001; Schnippenkoetter *et al.*, 2001; Willment *et al.*, 2002).

The similarities between the newly determined sequences and previously described MSV isolates allowed us to deduce that they contained all genomic features that have previously been identified as having functional relevance during MSV infections.

### Evidence of extensive inter-strain MSV recombination

**Fig. 1. f1:**
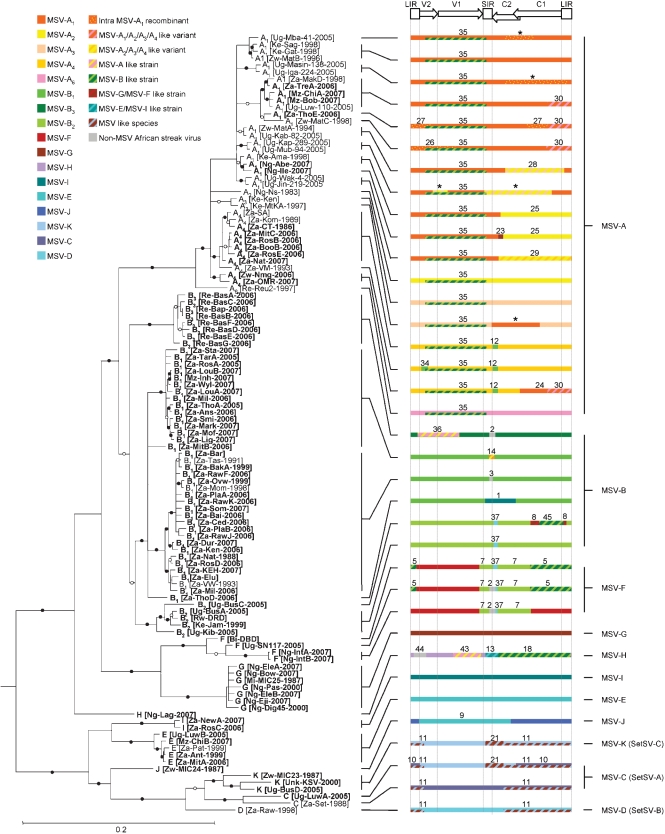
Complex relationships amongst MSV isolates sampled throughout Africa and the Indian Ocean island of La Réunion. Whereas tree branches with less than 50 % bootstrap support have been collapsed, those with greater than 70 and 90 % support are labelled with open and filled-in circles respectively. The tree was rooted using *Panicum* streak virus (isolate Karino; GenBank accession no. L39638) as an outgroup (not shown). Virus names take the form Strain_variant_ [country–region–laboratory ID–year of isolation]. Variant numbers are equivalent to the subtype designators given in other publications (Owor *et al.*, 2007b; Martin *et al.*, 2001). Wherever representation of older recombination events would have obscured the representation of more recent recombination events, the older events have been displayed as a thinner bar. Hatched regions indicate tracts of sequence either transferred from currently unsampled MSV strains during a relatively recent recombination event, or transferred between ancestral sequences during a more ancient recombination event. Numbers associated with recombination events correspond with those in Supplementary Table S3. Events marked with an asterisk were characterized in Owor *et al.* (2007b). Positions of genomic features are indicated above the coloured bars: V2, movement protein gene; V1, coat protein gene; C1/C2, replication-associated protein gene; C1, *repA* gene; LIR, long intergenic region; SIR, short intergenic region. Bar, 0.2 nucleotide substitutions per site.

As recombination features prominently in geminivirus evolution (Lefeuvre *et al.*, 2007b; Martin *et al.*, 2001; Padidam *et al.*, 1999; Prasanna & Rai, 2007) and can cause phylogeny reconstruction errors (Awadalla, 2003; Penny *et al.*, 2007; Posada & Crandall 2002) we tried to remove, as far as possible, the influence of recombination from the construction of an MSV phylogeny. We therefore analysed the 83 newly sequenced genomes together with all other 112 publicly available African streak virus genomes using a battery of seven recombination analysis methods implemented in the rdp3 program. We found clear evidence of 36 distinct recombination events (detectable by three or more different analysis methods and with good phylogenetic support) spread across 164 of the 195 analysed genomes. Twenty-seven of these events were detected in 157 of the 172 analysed MSV sequences (Fig. 1; Supplementary Table S3 and Supplementary rdp3 project file).

Due to such a high proportion of the analysed MSV sequences being detectably recombinant (91.3 %), it proved more difficult to produce a recombination-free MSV phylogeny than we had anticipated. The primary problem was that there was no fraction of the sequence alignment longer than 519 nt (alignment positions corresponding to nt sites 311 and 760 in MSV-Ns, taking position 1 as the first A residue 3′ of the virion strand origin of replication) that was unbroken by detectable recombination breakpoints in any of the sequences. As this 519 nt region is quite conserved amongst the MSV isolates, phylogenetic analyses focusing on it lacked sufficient power to resolve relationships amongst individuals within particular strains (see Supplementary Fig. S2 for this recombination-free phylogeny).

To provide a general description of the relationships between all of the sequences we therefore opted to simply construct a maximum-likelihood tree using the full genome sequences, essentially ignoring recombination, and present it together with a breakdown of the sequence mosaics that must be considered when interpreting its topology (Fig. 1).

Despite recombination undermining our confidence in the accuracy of this MSV phylogeny, there is good bootstrap support (>70 %) for all of our tentative MSV strain classifications. It should, however, be pointed out that the recombination analysis indicated that only four of the 11 strains (MSV-B, MSV-E, MSV-G and MSV-I) are predominantly represented by sequences that are not the products of inter-strain recombination events involving exchanges of more than 30 % of their genomes.

Some of the recombinant strains, such as MSV-H and -F, appear to have quite complex mosaic structures. For example, Ng-Lag-2007, the only MSV-H isolate we have sampled, has a genome that appears to have been assembled during at least four separate recombination events. Adding to the complexity of interpreting the origins of sequences such as Ng-Lag-2007 is that we cannot know, without better sampling, either how old many of these recombination events are, or the order in which they most likely occurred. For example, lack of clear evidence for where the different pieces of Ng-Lag-2007 have come from indicates that the detected recombination events occurred between either progenitors of the sampled strains (i.e. if they are older events), or divergent, currently unsampled MSV genotypes (i.e. if they are more recent events).

Possibly the most interesting amongst the less complex recombinant strains is MSV-A – the strain that causes maize streak disease. Previous analyses of recombination amongst MSV strains (Martin *et al.*, 2001; Padidam *et al.*, 1999) failed to detect that this strain had arisen from an ancient recombination event between MSV-G/MSV-F and MSV-B progenitors. This was because without the newly discovered MSV-G and -F genomes it was not possible to tell that the MSV-A virion sense ORFs were unusually similar to those of the MSV-Bs. Every currently sampled MSV-A genome has an unmistakable trace of this recombination signal (including the most divergent genomes from La Réunion; *P*=7.4×10^−9^), indicating that the recombination event must have occurred prior to the time of the last common ancestor of all known MSV-As.

We must, however, caution that, given the inherent difficulties associated with identifying recombinant sequences in datasets with such high degrees of recombination, it is possible that we have misidentified MSV-A as the recombinant in this sequence exchange. That a recombination event has occurred is very probable, but we cannot be absolutely certain that it is not all of either the MSV-B or MSV-G and MSV-F sequences that are recombinant instead of the MSV-A sequences. It may require either the discovery of non-recombinant close relatives of the MSV-A viruses or fitness studies on laboratory reconstructions of the possible ancestral parental and recombinant viruses to prove that it is MSV-A and not the other strains that are recombinant. However, until this information becomes available, the most parsimonious hypothesis presented by RDP3 is that the MSV-As are recombinant.

### Partial conservation of recombination patterns amongst geminiviruses

**Fig. 2. f2:**
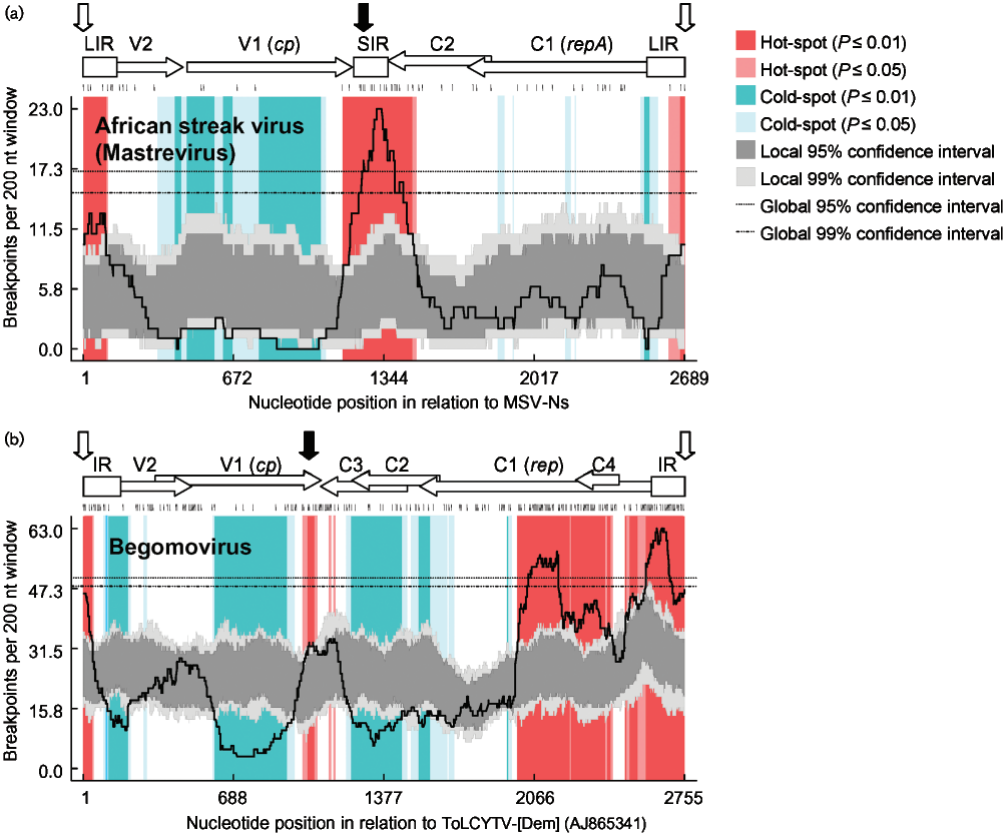
Partial conservation of recombination breakpoint distributions across the family *Geminiviridae*. (a) Breakpoint distribution plot (solid black line) indicating recombination hot- and cold-spots detectable in African streak virus sequences. Broken lines represent 99 and 95 % confidence intervals for the ‘global’ hot-spot test of Heath *et al.* (2006). Light and dark shaded regions, respectively, represent 99 and 95 % confidence intervals of the ‘local’ hot- and cold-spot test of Heath *et al.* (2006). (b) Recombination breakpoint distribution plot for the begomoviruses (after Lefeuvre *et al.* 2007b). Positions of genomic features are indicated above the plots: horizontal arrows labelled V and C, respectively, represent virion and complementary sense genes; boxes labelled IR, LIR or SIR represent intergenic regions. *cp*, Coat protein gene; *rep*, replication-associated protein gene; *repA*, ORF encoding the N-terminal portion of Rep. Vertical black arrows indicate polyadenylation signals of the complementary-sense genes and white arrows indicate the virion-strand replication origin.

Conserved patterns of inter-species geminivirus recombination including recombination hot- and cold-spots have recently been described amongst members of the genus *Begomovirus* (Lefeuvre *et al.*, 2007a, b; Prasanna & Rai, 2007). Although similarities in intra-strain recombination rates have been demonstrated for MSV-A and variants of the begomovirus species, East African cassava mosaic virus and East African cassava mosaic Kenya virus (Owor *et al.*, 2007b), it is currently unknown whether patterns of inter-species/strain recombination are also conserved between begomoviruses and mastreviruses. We therefore analysed the distribution of breakpoints detected in our African streak virus dataset using the method described by Heath *et al.* (2006). Despite the relatively low number of unique recombination events analysed (36 in the African streak virus dataset compared with 284 collectively detected in the datasets analysed by Lefeuvre *et al.*, 2007b) there was strong statistical evidence of recombination hot-spots near the coat protein gene (*cp*)/short intergenic region (SIR) interface and at the virion-strand origin of replication (v-*ori*), and a recombination cold-spot spanning almost the entire *cp* (Fig. 2a[Fig f2]). Importantly, these hot- and cold-spots are in almost precisely the same locations as those detected previously in begomoviruses (Fig. 2b[Fig f2]). Unlike with the begomoviruses, however, in the mastrevirus genomes there is no evidence of a clear recombination hot-spot near the centre of the replication associated protein gene (*rep*). Also, in the mastrevirus dataset the *cp*/SIR interface hot-spot is substantially more pronounced than the v-*ori* hot-spot, whereas the converse is true for the begomoviruses.

Despite these differences, this result strongly suggests that similar processes are shaping recombination breakpoint distributions in both genera. Importantly, the observed recombination patterns, including the differences between them, are entirely consistent with recent hypotheses that have invoked a mixture of biochemical and selective forces to explain non-random recombination breakpoint distributions in geminiviruses (Jeske *et al.*, 2001; Lefeuvre *et al.*, 2007a, b).

In both the begomoviruses and mastreviruses the recombination hot-spots map to complementary-sense gene transcription initiation and termination sites and virion-strand origins of replication. The reason complementary gene transcription initiation and termination sites may be more predisposed to recombination than other sites is possibly that these are the regions where the most frequent clashes between transcription and replication complexes occur (Lefeuvre *et al.*, 2007a). The absence in mastreviruses of a transcription initiation site and promoter elements in the middle of *rep* analogous to those found in begomoviruses (Shung *et al.*, 2006) may explain why there is no detectable recombination hot-spot in this region of mastrevirus genomes.

It is also possible that the absence of a short intergenic region in begomoviruses could be the reason for the recombination hot-spot mapping to the 3′ end of *cp* in begomoviruses being smaller than that detected in mastreviruses. The distribution of recombination breakpoints detectable in our mastrevirus dataset is consistent with proposals that breakpoint distributions observed in geminiviruses sampled from nature are strongly influenced by selective forces that eliminate recombinants with defective intra-genome interactions (Martin *et al.*, 2005c; Lefeuvre *et al.*, 2007b). It has been convincingly demonstrated that selection strongly favours the survival of geminivirus recombinants in which both intra-protein amino acid interactions (Lefeuvre *et al.*, 2007b) and inter-genome region interactions (Martin *et al.*, 2005c) remain undisrupted. Importantly, there are various lines of evidence that indicate that recombination breakpoints both at the 3′ end of *cp* (García-Andrés *et al.*, 2007a; Lefeuvre *et al.*, 2007b) and within the SIR (Martin *et al.*, 2005c; Martin & Rybicki, 2002) are particularly undisruptive of intra-genome interactions. MSV SIR sequences are extremely modular and can continue functioning properly even when transferred into genetic backgrounds very different from those in which they evolved (Martin & Rybicki, 2002). If the modestly sized recombination hot-spot at the 3′ end of the begomovirus *cp* is caused by the coincident location of a site encoding a proportion of CP that tolerates recombination well at the same position as a transcription terminator that is biochemically predisposed to recombination (due to clashes between transcription and replication complexes), then it is reasonable to suspect that the placement of a highly modular intergenic region beside this site in mastreviruses is responsible for the larger size of this hot-spot in these viruses.

### Differences in the geographical distributions of MSV strains and variants

**Fig. 3. f3:**
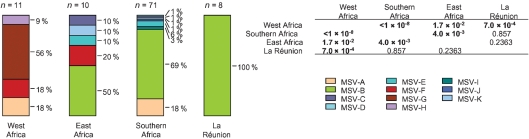
MSV strain demographics in different parts of Africa and La Réunion. All virus isolates represented here were sampled from uncultivated grass species. Different strains are represented by different colours, and *P* values indicate regions with significantly different MSV-population structures (values in bold).

We were interested in determining whether there were differences in MSV strain demographics in different parts of Africa analogous to those previously detected for cassava-infecting geminiviruses (Ndunguru *et al.*, 2005; Bull *et al.*, 2006). We therefore split the sampled viruses into southern African (isolates from Zimbabwe, South Africa and Mozambique; *n*=70), West African (isolates from Nigeria and Mali; *n*=11), East African (isolates from Uganda, Rwanda, Burundi and Kenya; *n*=10) and La Réunion (*n*=8) groups and tested for differences in the strain compositions of these groups. Although there was a highly significant difference in the strain distributions across all four regions collectively (*P*=8.3×10^−8^, 4 [regions]×11 [MSV strains] *χ*^2^ test), separate pairwise comparisons between the regions indicated that these differences originated primarily from the West African population sample (Fig. 3). While the East African and southern African strain distributions were also significantly different, the West African strain distribution is clearly the most unique. The key differences between the sampled West African MSV population and those found elsewhere are the absence of any MSV-B isolates and the presence of MSV-G and -H isolates.

This was a surprising result as we had anticipated that the mainland African MSV-populations would have similar structures, and that the La Réunion population would be distinct. Recent reports of large numbers of new geminivirus species, strains and variants unique to the Indian Ocean islands (Delatte *et al.*, 2005; Lefeuvre *et al.*, 2007a; Peterschmitt *et al.*, 1996; Shepherd *et al.*, 2008b) have indicated that they have been reasonably isolated with respect to the movement of geminiviruses. Conversely, the close relationships shared by MSV strain A isolates found in West Africa with those found elsewhere on the continent (Briddon *et al.* 1994; Martin *et al.*, 2001) clearly indicate that the movement of at least some MSV strains either to or from West Africa is relatively frequent and largely unhindered. Our failure to sample any MSV-B isolates in West Africa and any MSV-G and -H isolates outside West Africa therefore suggests that there may be strain-specific differences in the continent-wide movement of MSV variants.

**Fig. 4. f4:**
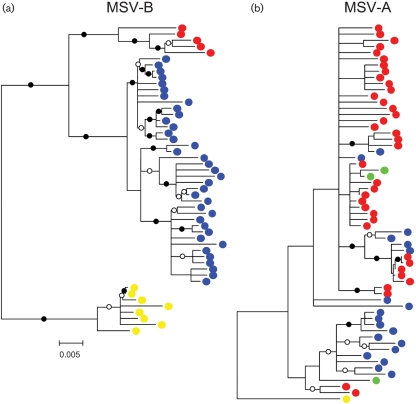
Phylogenetic evidence that MSV-A variants move between and become established in different regions of Africa more frequently than MSV-B variants. (a) Relationships amongst MSV-B sequences from wild and cultivated grass species in East Africa (red circles), southern Africa (blue circles) and La Réunion (yellow circles). (b) The relationships amongst MSV-A isolates from wild grasses and maize sampled in West Africa (green circles), East Africa (red circles), southern Africa (blue circles) and La Réunion (yellow circles). Both trees were constructed using sequence alignments from which tracts of recombinationally derived sequences were deleted (i.e. they are largely recombination free). Also in both trees, branches with less than 50 % bootstrap support have been collapsed and branches with 70 (○) or 90 % (•) bootstrap support are labelled. Bar, 0.005 nucleotide substitutions per site.

To investigate this possibility further we compared the phylogenies of MSV-A and -B isolates (the two best sampled MSV strains) in the context of their regions of origin (Fig. 4). Both MSV-A and -B isolates from particular regions tend to group in phylogenetic trees with other isolates from the same region. However, all MSV-B isolates from particular regions form monophyletic groups (i.e. all viruses from, for example, southern Africa are more closely related to other southern African viruses than they are to East African or Réunion viruses), which is not the case for the MSV-A isolates. MSV-A isolates sampled in different parts of Africa are polyphyletic in that, for example, different groups of southern African isolates are more closely related to West and East African isolates than they are to other groups of southern African isolates. This difference between the MSV-A and -B phylogenies strongly suggests that, over the evolutionary timescales represented by these trees, MSV-A variants are moving between and becoming established in different parts of Africa at a greater rate than MSV-B variants. This implies that there are ecological barriers to the movement of MSV-B variants across Africa that are not experienced by MSV-A variants and provides good support for our suggestion that there are strain-specific differences in the continent-wide movement of MSV variants across Africa.

Given that the MSV-A, -B and -C strains all share common vector species with other African streak virus species (Schnippenkoetter *et al.*, 2001; Willment *et al.*, 2002; Bigarré *et al.*, 1999; Briddon *et al.*, 1992) it would be reasonable to suppose that all of the other newly discovered strains also probably share the same vector species. It might therefore seem obvious that strain specific differences in host ranges and/or degrees of host adaptation are probably responsible for differences in their geographical distributions. It cannot, however, be ruled out that the different MSV strains are specifically adapted to transmission by different vector races or biotypes (Mesfin *et al.*, 1991) and that differences in the feeding preferences and geographical distributions of these races and biotypes might underlie differences in MSV strain distributions.

Also, while we have dealt here with MSV isolates sampled from uncultivated grasses, it is important to point out that the distributions of cultivated MSV host species such as maize, wheat, sugar cane and millet might also have an important impact on the continental spread of different MSV strains. For example, a major host of MSV-A is maize and the widespread distribution of this cultivated species has possibly aided the movement of MSV-A throughout the continent. Another possibility that should be considered with crop-infecting viruses such as MSV is that they might be transported directly by humans. MSV-A isolates have recently been identified infecting sugar cane throughout large parts of South Africa (van Antwerpen *et al.*, 2008). As infections are characteristically mild and sugar cane is vegetatively propagated, it is possible that inadvertent transportation of infected stalks might accelerate the movement of MSV-A variants. It is even conceivable that transportation of MSV-A-infected maize cobs might facilitate the movement of this strain. Although MSV is not seed transmitted, immature maize cobs are frequently transported within their leaf-like sheaths. These ‘green envelopes’ display streak symptoms in MSV-infected plants and the virus could therefore presumably be acquired by leafhoppers should they feed on them. However, good phylogenetic evidence of at least some MSV-A diversification along geographical lines indicates that if long-distance human transportation of MSV-A across the continent occurs at all, it is probably infrequent.

### Host range variation amongst MSV strains

In an attempt to directly determine whether differential host preferences (either by virus strains or vector biotypes) might at least partially account for differences in the geographical distributions of different strains, we analysed the strains in the context of the hosts from which they were isolated. Grouping hosts by genus (11 groups excluding cultivated host species) and viruses by strain (10 groups – excluding MSV-D for which no clear host identification could be made) we found very strong statistical support (*P*<1×10^−8^; 11×10 *χ*^2^ test) for significant differences between the hosts from which members of different MSV-strains were sampled.

However, we noted that we had oversampled certain host genera (e.g. *Digitaria* species, *n*=39) and undersampled others (e.g. *Axonopus* species, *Rottboellia* species and *Pennisetum* species, *n*=1 each; Supplementary Fig. S3 available in JGV Online). We also realized that on the island of La Réunion we had apparently oversampled *Cenchrus* species relative to other regions (3/4 of all *Cenchrus* samples were obtained on this island). Importantly, we found evidence of significant differences between the host types sampled in different regions [*P*=0.027, 10 (host genera)×4 (geographical region) *χ*^2^ test]. This sampling bias was primarily accounted for by the greater numbers of *Cenchrus* species sampled from La Réunion as it could be resolved by removing the La Réunion sample from the analysis (*P*=0.149). Given that only MSV-B isolates were sampled on La Réunion and that the sampling bias was only marginally significant, we did not anticipate that it would have a substantial effect on our assessment of the prevalence of different MSV strains in different host species. It is important to point out, however, that our sampling of obviously symptomatic plants may have unpredictably biased our analysis of natural host range distributions, in that unsampled host species/MSV strain combinations associated with mild or asymptomtic infections might be just as epidemiologically relevant as those associated with severely symptomatic infections.

**Fig. 5. f5:**
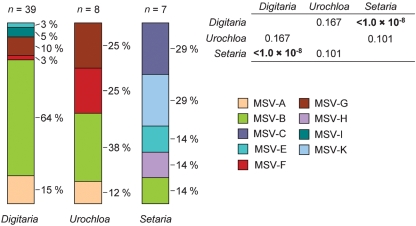
The frequencies with which different MSV strains were sampled from three common groups of MSV hosts. Different strains are represented by different colours and *P* values indicate significant differences (values in bold) between the three groups with respect to the MSV strains isolated from their members.

We sought to offset some of these potential sampling biases by investigating the frequencies with which different MSV strains were sampled only in the three host genera for which we obtained seven or more samples: *Digitaria* species (*n*=39), *Urochloa* species (*n*=8) and *Setaria* species (*n*=7; Fig. 5). We found significant differences in the MSV strains isolated from the plants of these three genera [*P*<1×10^−8^, 3 (host genera)×9 (virus strains−no MSV-D and -J isolates were sampled from the three genera considered) *χ*^2^ test]. Considering the host genera in pairs we only found a significant difference between the relative frequencies with which different MSV strains were sampled from *Digitaria* and *Setaria* plants (*P*<1×10^−8^). Whereas *Setaria* plants tended to be infected with the closely related MSV-K and -C strains, *Digitaria* plants tended to be infected with the closely related MSV-A, -B and -G strains.

We then looked for specific differences between the apparent host ranges of different virus strains. Despite our sampling too few viruses to achieve enough statistical power to differentiate between the host ranges of most of the MSV strains, we observed significant natural host range differences between MSV-B and both MSV-C and -K [*P*=1.0×10^−4^ and 2.0×10^−4^, respectively; 2 (virus strains)×11 (host genera) *χ*^2^ test] and between MSV-G and both MSV-C and -K (*P*=2.9×10^−2^ and 5.0×10^−2^, respectively). Whereas MSV-B and -G isolates tended to come primarily from *Digitaria* species, MSV-K and -C isolates tended to come primarily from *Setaria* species. Should the MSV-C, -D and -K strains (Fig. 1) be separated into another species following the next revision of geminivirus taxonomic criteria, it seems logical, therefore, that the species be named *Setaria* streak virus and that these strains be renamed as SetSV-A, -B and -C, respectively (Fig. 1).

Although there was no significant difference in the range of host genera from which MSV-A and -B isolates were sampled (*P*=0.28), we noted that the 14 MSV-A samples were obtained from grasses in eight genera, whereas the 34 MSV-B samples were obtained from grasses in only six genera. While this may indicate that MSV-A has a broader host range than MSV-B – something that may explain differences in the continent-wide distribution of the strains – more intensive sampling in a greater variety of hosts will be required to conclusively prove this. Nevertheless, our discovery of MSV-A isolates infecting a variety of uncultivated grasses is significant in that these species are probably both the natural hosts from which this strain originally emerged as a maize pathogen, and represent the hosts that currently sustain it between maize growing seasons.

### Conclusions

While our analysis of MSV diversity in uncultivated indigenous African grasses has revealed six new MSV strains, we have also detected for the first time, a degree of recombination amongst mastreviruses paralleling that seen in begomoviruses. Our demonstration that recombination patterns are partially conserved across the family *Geminiviridae* is particularly significant in that it indicates that early ancestral geminivirus genomes had largely the same recombinational predispositions and constraints as those experienced by modern geminiviruses. Of potentially greater immediate relevance, however, is our discovery that the maize-adapted MSV-A strain is possibly the product of an ancestral recombination event between *Digitaria*-adapted MSV-G/-F and MSV-B viruses. While this recombination event may have produced a virus with increased severity in maize – a host to which MSV-A seems particularly well adapted – our data are also consistent with the possibility that it may have enabled MSV-A to spread more efficiently throughout the continent by allowing it to infect a wider variety of hosts. Importantly, these hypotheses can be directly tested by reconstructing and analysing the virulence and host ranges of the ancestral MSV-A and its parental viruses. Despite the amount of speculation about how recombination may produce new viral species or strains with altered host ranges, cell tropisms or pathogenicities, there are actually very few well supported examples of this having occurred in nature (see Fondong *et al.*, 2000; Pita *et al.*, 2001; Monci *et al.*, 2002; García-Andrés *et al.*, 2007a, b for good exceptions). The possibility that a recombination event is ultimately responsible for the existence of maize streak disease certainly deserves thorough investigation as MSV-A might be an important example of how recombinational acquisition of novel traits can sometimes trigger pathogen emergence.

## Supplementary Material

[Supplementary Material]
