# Supplementary material for: Recombination, decreased host specificity and increased mobility may have driven the emergence of maize streak virus as an agricultural pathogen
Source: J Gen Virol. 2008 Sep;89(Pt 9):2063–74. doi: 10.1099/vir.0.2008/003590-0 (PMC2886952; doi:10.1099/vir.0.2008/003590-0)
Supplement: [Supplementary Material] [file supp_89_9_2063__index.html]

 Recombination, decreased host specificity and increased mobility may have driven the emergence of maize streak virus as an agricultural pathogen -- Varsani et al. 89 (9): 2063 Data Supplement - Supplementary Material -- Journal of General Virology

## 

### Recombination, decreased host specificity and increased mobility may have driven the emergence of maize streak virus as an agricultural pathogen, by A. Varsani, D. N. Shepherd, A. L. Monjane, B. E. Owor, J. B. Erdmann, E. P. Rybicki, M. Peterschmitt, R. W. Briddon, P. G. Markham, S. Oluwafemi, O. P. Windram, P. Lefeuvre, J.-M. Lett and D. P. Martin

*Journal of General Virology* vol. **89**, part 9, pp. 2063 - 2074

**Supplementary Figures**  [PDF]  (87 KB)

**Supplementary Table S1**  XLS file (32 KB)

**Supplementary Table S2**  XLS file  (383 KB)

**Supplementary Table S3**  XLS file  (71 KB)

**Supplementary data**  RDP file  (1542 KB)

  
  
