# Supplementary material for: Recombination, decreased host specificity and increased mobility may have driven the emergence of maize streak virus as an agricultural pathogen
Source: J Gen Virol. 2008 Sep;89(Pt 9):2063–74. doi: 10.1099/vir.0.2008/003590-0 (PMC2886952; doi:10.1099/vir.0.2008/003590-0)
Supplement: [Supplementary Material] [file supp_89_9_2063__1.pdf]

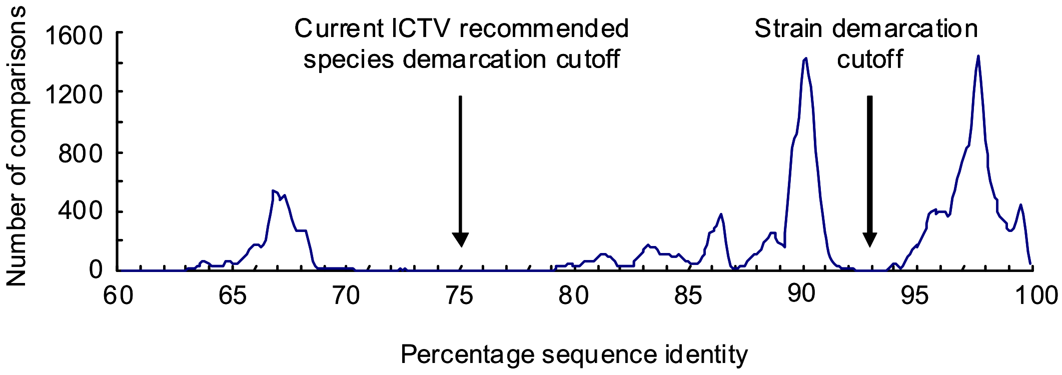

Supplementary Fig. S1. Rationalization of using 93% sequence identity as a strain demarcation criterion for African streak viruses. The blue line plots the frequencies of sequence identities shared amongst 357 African streak virus isolates at a resolution of 0.1% identity. The line was smoothed over a window of 0.3% identity. Identity values were calculated with pairwise exclusion of alignment gaps (as opposed to counting gaps as a fifth state as is currently done by the ICTV).

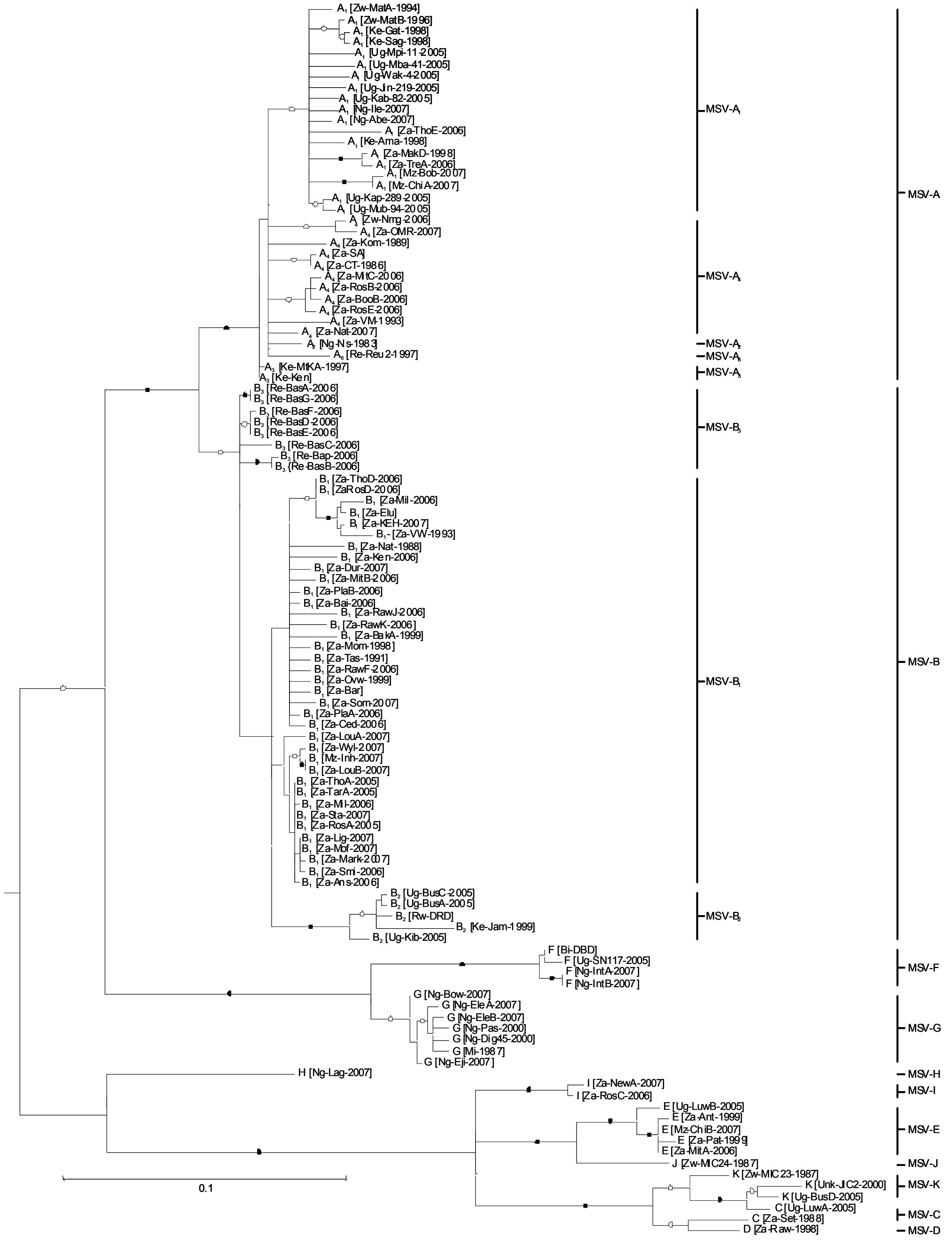

Supplementary Fig. S2. Maximum-likelihood tree (F81+G_4 model) constructed using a 519 bp long alignment of MSV genomes corresponding to MSV-Ns (referred to here as MSV A_2[Ng-Ns-1983]) nt coordinates 311–760 (where position 1 is the first A nucleotide 3′ of the virion-strand origin of replication). The significance of this genome fragment is that it is unbroken by detectable

recombination breakpoints and the presented tree is therefore mostly free of the influences of recombination. Branches in the tree with greater than 90% bootstrap support are labelled with a filled-in circle, branches with between 70 and 89% bootstrap support are labelled with an open circle, and branches with less than 50% bootstrap support have been collapsed. The tree was rooted using Panicum streak virus (isolate Karino; GenBank accession no. L39638) as an outgroup (not shown). The 11 MSV strains (MSV-A through -K) and MSV-A (1–4 and 6) and MSV-B (1–3) variant groups are indicated on the left.

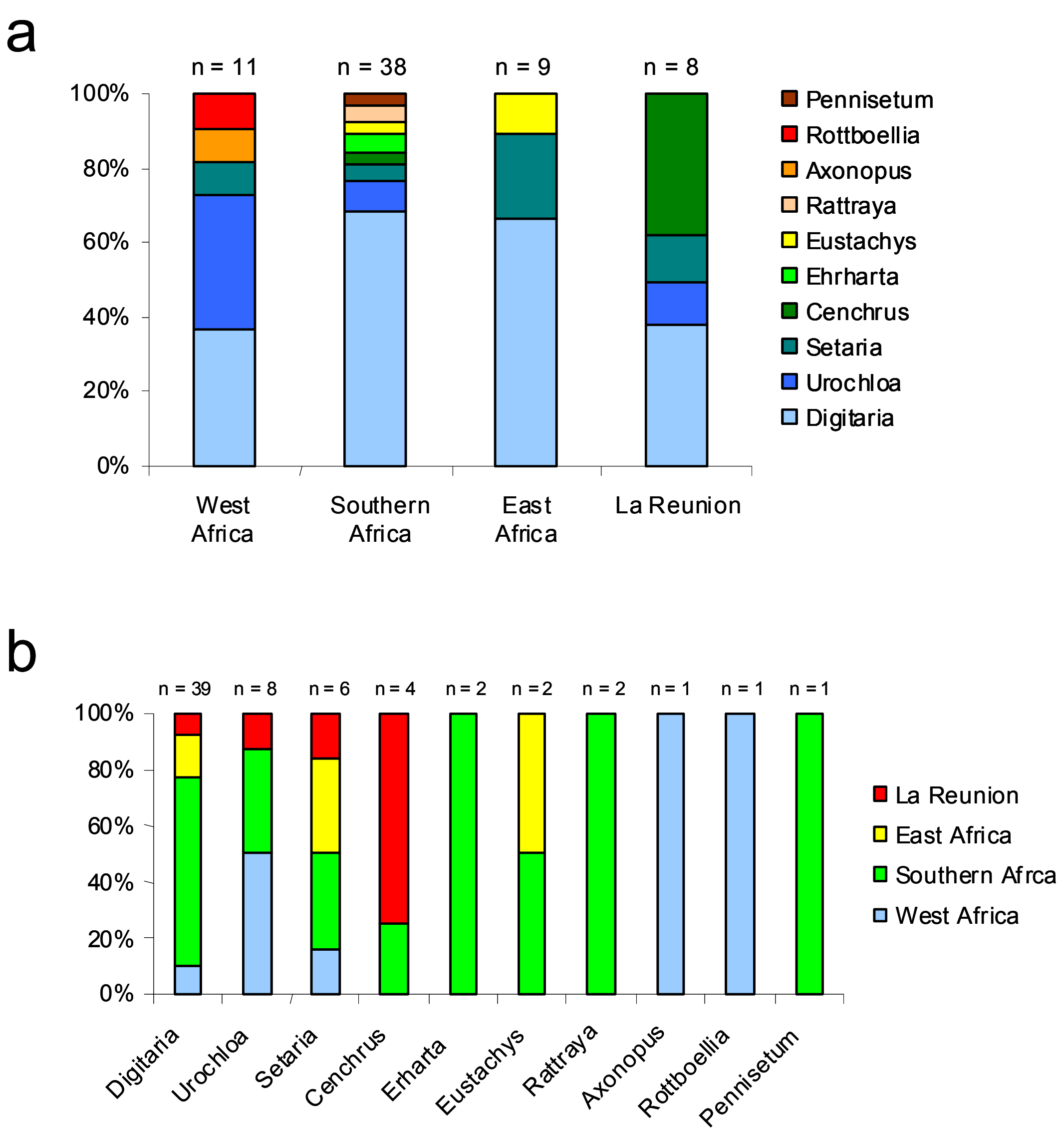

Supplementary Fig. S3. Geographical distribution of sampled grass species. (a) Distribution of species found in different sampling zones. (b) Geographical origins of different grass species.
